# Percutaneous Trans-septal Mitral Valve-in-Ring Implantation Using a Transcatheter Balloon-Expandable Transcatheter Heart Valve With Elective Intra-Procedural Artero-Venous ECMO in a Patient With Severely Reduced Left Ventricular Ejection Fraction

**DOI:** 10.3389/fcvm.2019.00174

**Published:** 2019-12-04

**Authors:** Francesca Ristalli, Silvia Maiani, Brunilda Hamiti, Alessio Mattesini, Francesco Meucci, Miroslava Stolcova, Carlo Di Mario

**Affiliations:** Structural Interventional Cardiology, Cardio-Toraco-Vascular Department, Careggi University Hospital, Florence, Italy

**Keywords:** mitral valve in ring, VA-ECMO, transcatheter heart valve (THV), trans-septal access, left ventricular dysfunction (LV dysfunction)

## Abstract

Percutaneous mitral valve-in-valve implantation is an emerging option in patients with surgical bioprosthesis failure or failing mitral annuloplasty and increased surgical risk. We present a case of transcatheter transvenous trans-septal mitral valve-in-ring (TMVinR) procedure, in a patient with severe left ventricular dysfunction and severe mitral regurgitation, after surgical mitral annuloplasty, managed with periprocedural mechanical circulatory support (MCS) with VA-ECMO.

## Introduction

Mitral valve repair and replacement are both feasible options in the treatment of functional mitral regurgitation (MR), with replacement demonstrating lower MR recurrence rate but not significant difference in left ventricular remodeling or survival at 12 months (1). Mitral valve repair is often the preferred option due to lower perioperative mortality (2, 3); an undersized ring annuloplasty is usually part of the repair procedure. Failure of the repair with recurrence of MR ≥ 2 grade was observed in nearly 27% of the patients at 10 years follow-up in a recently published study (4). Surgical re-intervention is seldom performed due to the high risk of mortality and morbidity, especially in elderly patients or in the presence of significant co-pathologies. Transcatheter valve replacement techniques have developed in the last years as a less-invasive alternative, particularly attractive for this high-risk population.

Transcatheter valve implantation in mitral position has been performed mainly in degenerated surgical bioprostheses but also in surgical rings, with good results (5, 6).

During the initial experience, mitral valve-in-valve procedures were performed almost exclusively using a trans-apical access, with the Sapien XT aortic valve (Edwards Lifesciences, Irvine, CA) mounted in the opposite direction on its delivery balloon (7). More recently, transvenous trans-septal mitral valve implantation technique has been described (8).

We present a case of severe MR after surgical annuloplasty in a patient with very poor left ventricular function, treated by transcatheter transvenous trans-septal mitral valve-in-ring (TMVinR) transcatheter heart valve (THV) implantation, during mechanical circulatory support (MCS) with veno-arterial extra-corporeal membrane oxygenator (V-A ECMO). Written informed consent was obtained from the participant for the publication of this case report.

## Case Report

A 72-year-old man with a history of ischemic and valvular heart disease and severely reduced left ventricular ejection fraction (EF) came to our attention due to heart failure and recurrent sustained ventricular tachycardias. He had been treated 25 years earlier with surgical patch-plasty of the left main coronary artery due to unstable angina. After 20 years, due to cardiac arrest and angiographic documentation of triple vessel coronary artery disease (CAD), he underwent coronary artery bypass grafting (CABG), with left internal mammary artery (LIMA) to left anterior descending (LAD) and sequential saphenous venous graft to intermediate and the obtuse marginal branch, together with mitral valve annuloplasty with a complete ring (Carpentier-Edwards Physio II n 26) and aortic valve replacement (Carpentier-Edwards Magna n 25). A year after the latter surgery, in 2013, he presented severe MR recurrence with evidence of detachment of the posterior portion of the annuloplasty ring and underwent re-do mitral surgery with suture of the detached portion and tricuspid plasty (Kay procedure) and MAZE ablation of atrial fibrillation. At that time, an implantable cardioverter-defibrillator (ICD) was implanted.

**Figure 1:**
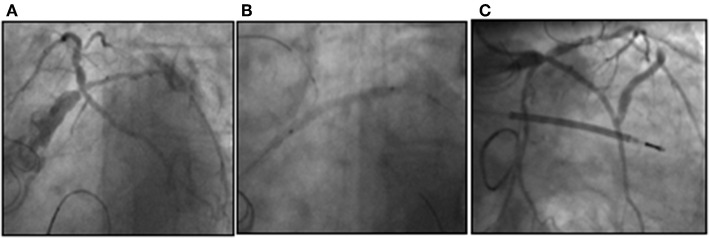
Critical stenosis of the distal LM involving the trifurcation with LAD, LCx, and intermediate branch **(A)**. A provisional approach was chosen to treat the LM bifurcation with sequential ballooning of RI and LCx **(B)** and the result after stent implantation in LM-RI and final kiss-balloon inflation **(C)**.

In September 2017, the patient was admitted to another institution because of acute heart failure and repeated syncopal episodes with documented ventricular tachycardia. A coronary angiogram showed critical left main and left circumflex artery (LCx) stenoses, venous graft occlusion, and patent LIMA graft. Transthoracic echocardiogram showed severe left ventricular dysfunction (EF 20–25%) and severe MR. An attempt for upgrading the ICD to CRT-D failed, because of insufficient coronary sinus dimensions. The patient was transferred to our institution for further care. The case was discussed in the Heart Team and the decision was made to treat the patient percutaneously with PCI and trans-catheter therapy of the MR, considering the prohibitive surgical risk (STS PROM 27.8%) of a 4th re-do surgery. The patient underwent PCI to left main coronary artery and intermediate branch (provisional approach to the bifurcation with LCx). The procedure was uncomplicated with good angiographic result (Figure 1).

**Figure 2:**
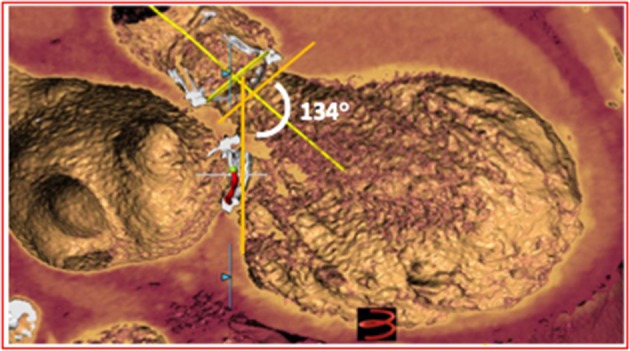
CT reconstruction showing the relationship between aortic and mitral bioprostheses. The aorto-mitral angle of 134° is associated with a low risk of LVOT obstruction.

Transesophageal echocardiography (TEE) was performed to better assess mitral valve anatomy. It showed severe tethering of both leaflets, large coaptation depth (12 mm), annular diameters of 14 × 22 mm and a mean trans-mitral gradient of 5 mmHg. Considering the echo data, the patient was deemed to be a poor candidate for a transcatheter edge-to-edge mitral valvuloplasty. An ECG-gated contrast-enhanced CT scan of the patient's heart and a CT angiogram of the thoraco-abdominal aorta were obtained in order to evaluate the pertinent anatomy for a valve-in-ring procedure. Cardiac CT scan was analyzed using a commercially available software (3Mensio Structural Heart Mitral Workflow, Pie Medical Imagining Maastricht, Netherlands) showing an annulus area of 2.8 cm^2^ and a perimeter of 62.2 mm. The measurements were consistent with the data from the Mitral Valve in Valve application (B.V. Mitral Valve in Valve Mitral app, http://www.ubqo.com/vivmitral) suggesting the use of an Edwards Sapien 3 23-mm valve. No dehiscence of the valvuloplasty ring was detectable on CT scan. The risk of left ventricular outflow tract (LVOT) obstruction was estimated to be low, based on large ventricular dimensions and absence of other LVOT obstruction predictive features. In particular, neo-LVOT estimated area was well-above the described cutoff of 250 mm^2^, and the calculated aorto-mitral angle was not indicative of high risk (Figure 2).

After evaluation of the clinical and imaging data, the decision was made to perform a trans-catheter trans-septal mitral valve implantation using a Sapien 3 valve. We also decided to perform the intervention using MCS with VA-ECMO due to the electrical instability of the patient and the low EF. According to the CT angiogram, the ilio-femoral arteries were suitable to accommodate the arterial cannula of the ECMO.

**Figure 3:**
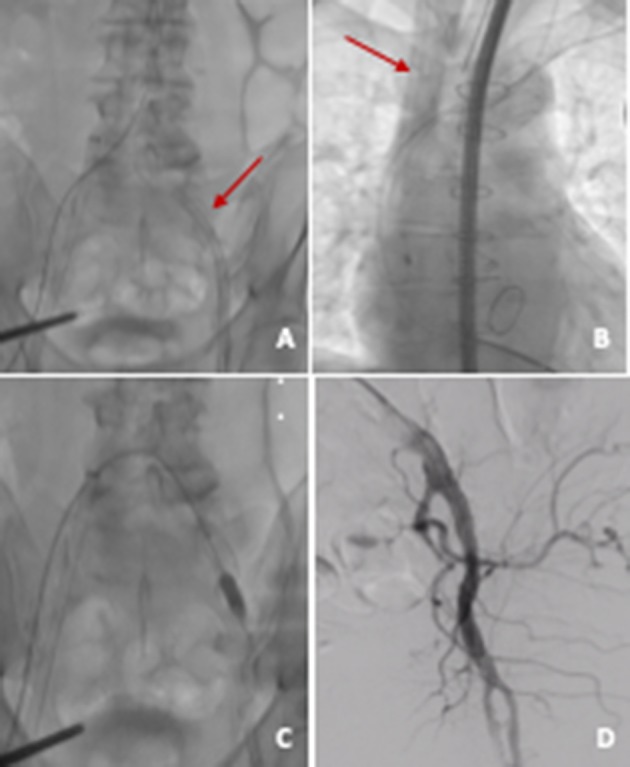
VA ECMO. **(A)** Arterial cannula (21 F) in left common femoral artery; **(B)** venous cannula (23 F) in right internal jugular vein; **(C)** arterial access closure with two preimplanted Proglides and intravascular hemostasis (10 × 20 mm peripheral balloon); **(D)** angiographic control showing good result on site of arterial access.

The procedure was performed under general anesthesia and TEE guidance. VA-ECMO was positioned via left femoral artery (21 F) and right jugular vein (23 F), with preimplantation of two Proglide devices (Abbott Vascular, Abbott Park, IL) on the arterial access. After positioning of the vascular cannulas, MCS was started with a flow rate of 2.1 L/min (Figure 3).

After obtaining right femoral vein access, trans-septal puncture was performed at the superior and posterior aspect of the fossa ovalis. The fossa was dilated with an 8.0 × 20 mm balloon and an 8F Mullins' sheath was placed in the left atrium. Then, the mitral valve was crossed using a 0.035″ hydrophilic wire supported by a 5F internal mammary artery catheter. The Mullins' sheath was advanced in the left ventricle, and two high-support pre-shaped guide wires (Safari S, Boston Scientific, Marlborough, MA) were positioned in the left ventricle to improve the support of the system. Further dilatation of the fossa ovalis was performed using a 14 × 40 mm peripheral angioplasty balloon and the Edwards 14F e-sheath was inserted in the right femoral vein. An Edwards Sapien 3 23-mm prosthesis was mounted with the skirt oriented toward the delivery handle for anterograde deployment in mitral position. Valve positioning on the delivery balloon was achieved in the inferior vena cava. The prosthesis was directed toward the mitral valve by full flexion of the Commander delivery system, in a fluoroscopic projection perpendicular to the plane of the ring.

**Figure 4:**
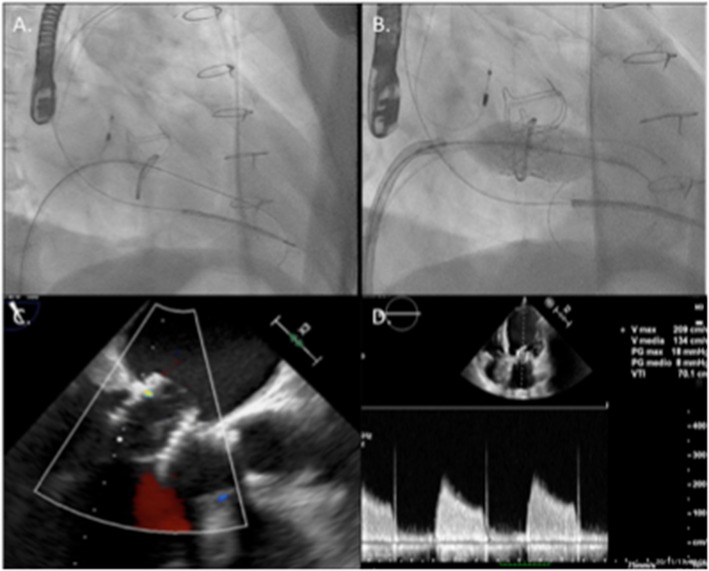
Main steps of the implantation procedure: in **(A)**, the mitral valve was crossed with a supportive pre-shaped wire and the Mullins' sheath. **(B)** Shows valve deployment under rapid pacing. **(C)** Shows final echographic result with the valve correctly positioned in the mitral ring with no significant leak and acceptable 5 mmHg transvalvular gradient **(D)**.

Subsequently, the prosthesis was placed within the mitral ring. Its correct position was carefully checked by fluoroscopy and TEE and it was deployed by slow balloon inflation under rapid pacing (180 bpm). Echocardiography revealed only trivial paravalvular leak, absence of LVOT obstruction and mean trans-mitral diastolic gradient of 5 mmHg (Figure 4). Interatrial septum presented small residual left to right shunt at the trans-septal puncture site.

During the procedure, especially after the crossing of the mitral valve with the stiff wires, the patient presented prolonged episodes of absence of pulsatile pressure and was completely dependent on ECMO circulatory support (flow rate 3.0 L/min, 3,000 revolutions/min). However, after THV implantation, the patient maintained hemodynamic stability and the VA-ECMO was removed in the cath lab, following rapid weaning. The arterial access was closed with the pre-implanted Proglides, without complications. The patient was transferred to the referring hospital after 5 days.

At 6 months follow-up, the patient presented with improved general conditions, with moderate effort dyspnea, while on Furosemide 125 mg bid. Transthoracic echocardiography showed the prosthesis in a correct position with mild paravalvular leak and a mean transvalvular gradient of 5 mmHg. Left ventricular EF was 20%.

## Discussion

Transcatheter mitral valve replacement is gaining success as an alternative to surgery in high-risk patients presenting with degeneration of bioprostheses in mitral position or failing mitral annuloplasty rings (6, 7). Previous reports have described the feasibility of implantation of transcatheter prostheses in surgical annuloplasty rings (8), despite the theoretical limitations related to the asymmetrical shape, size, and deformability of the ring.

Pre-procedural planning is of paramount importance in this setting. First of all, information on the previous surgery and the underlying mitral valve disease needs to be as complete as possible.

There are multiple types of rings available for surgical mitral repair, classified in terms of flexibility, geometry, and circumferential completeness. The presence of a complete or incomplete ring is an important factor to consider during pre-procedural planning and an accurate CT-scan evaluation is crucial to investigate and confirm structural features of the ring. Physio II Carpentier Edwards ring is a rigid, complete, saddle-shaped ring. Compared with semi-rigid or flexible rings, rigid ones may retain their original D-shape even after THV implantation, carrying a risk of incomplete paravalvular sealing. Slightly oversizing the THV and high-pressure post-dilatation can minimize PVR in cases of TMVInR (9). Also, the type of valve pathology (stenosis or regurgitation) must be taken into account for a correct sizing.

Transoesophageal echocardiography allows the assessment of valvular dysfunction severity and the mechanism of annuloplasty failure, including the exclusion of a coexistent annular dehiscence. A dedicated ECG-gated contrast-enhanced CT scan is useful for the sizing of the prosthesis. Even if the information about the previously implanted surgical ring is available, CT imaging is still invaluable to confirm annular shape and dimensions as well as to predict LVOT obstruction risk, especially in valve-in-ring procedures where the native anterior mitral valve leaflet might be freely displaced toward the LVOT by the percutaneous prosthesis. Several predictors of LVOT obstruction, including septal hypertrophy, a small left ventricular cavity, an aorto-mitral annular angle of <135°, and a length of the anterior MV leaflet (AML) >30 mm have been identified (10, 11). To avoid the latter cause of LVOT obstruction, Babaliaros et al. (12) have proposed, prior to THV implantation, a percutaneous splitting of the anterior mitral leaflet by means of an electrified guide wire that traverses the leaflet base, between two retrograde aortic catheters, and which is then pulled outwards toward the leaflet tip to cause its tear. Use of this technique has been reported in small numbers of cases with promising results (12).

Commonly aortic THVs are used in mitral position for TMVinR procedures, namely, Edwards Sapien valves and the Lotus valve. In addition to CT scan measurements, a Mitral Valve in Valve application is available (B.V. Valve in Valve Mitral app, http://www.ubqo.com/vivmitral), providing helpful sizing suggestions for valvuloplasty rings as well.

After defining THV size, a simulation of its positioning inside the ring can be done, considering to deploy 80–90% of the THV into the LV and 20–10% into the left atrium. This allows a visual measurement of procedural result and a direct measure of the residual neo-LVOT area for LVOT-obstruction risk prediction. A cutoff Neo-LVOT area predicting a significant risk for LVOT obstruction is not well-defined at this moment, but some studies showed that a Neo-LVOT of 250 mm^2^ or more have a lower risk of LVOT obstruction (8). Another important factor to consider in valve positioning is the valve flaring at the ventricular end, which could reduce the risk of delayed migration of the THV, but also contribute to the LVOT obstruction. The degree of flare is directly proportional to the degree of LVOT area reduction (10).

Recently, the transvenous trans-septal approach has emerged as an alternative to trans-apical approach and appears to be less invasive and preferred by patients although more technically challenging. Preliminary data from the VIVID registry showed more pronounced improvement in left ventricular EF and possibly lower mortality (13, 14). A reported experience of 521 patients undergoing transcatheter mitral valve implantation (322 ViV, 141 VinR, 58 valve in native mitral valve with calcified annulus—VinMAC) provided excellent outcomes for patients with degenerated bioprostheses; VinR and VinMAC were associated with higher rates of adverse events and mid-term mortality compared to ViV, due to more frequent complications, in particular post-procedural MR and LVOT obstruction (15). However, need for a second valve decreased with experience and was not associated with worse outcomes. Patients treated with VinR experienced significant improvement of symptoms, suggesting that this technique is a reasonable alternative for high-risk patients.

Considering the patient's clinical conditions, particularly the severe EF reduction, we considered the patient to be at high risk of intra-procedural hemodynamic instability. We therefore elected for a peri-procedural percutaneous implantation of VA-ECMO. This device was selected due to the absence of interference with aortic valve, a bioprosthesis in our case, and peripheral positioning not requiring implantation of a device inside left cardiac chambers. In order to avoid interference with the valve prosthesis delivery system, we decided to position the venous cannula in right internal jugular vein, with the tip at the superior vena cava-right atrium junction level. VA-ECMO can provide high circulatory flow (3–7 L/m), warranting adequate hemodynamic support during invasive maneuvers determining cardiac output reduction and during the fast ventricular pacing required for valve implantation (16). We preferred this device to conventional extracorporeal circulation due to versatility and to the possibility of maintaining it after the procedure if needed. Due to the large dimensions of the arterial cannula, preprocedural peripheral vessels CT scan is essential to evaluate arterial dimensions in order to ascertain the feasibility of the 21 F cannula positioning.

## Conclusions

Percutaneous TMVinR implantation is safe and feasible with good acute and intermediate results. We suggest the use of MCS in patients at high risk of hemodynamic instability.

## Data Availability Statement

All datasets generated for this study are included in the article/supplementary material.

## Author Contributions

FR, AM, and FM has contributed to procedure planning, patient management, data collection and processing, and manuscript editing. SM has contributed to data collection and processing and manuscript editing. BH has contributed to patient management. MS has contributed to patient management and procedure planning. CD has contributed to procedure planning, patient management, and manuscript editing.

### Conflict of Interest

The authors declare that the research was conducted in the absence of any commercial or financial relationships that could be construed as a potential conflict of interest.
